# Supplementary material for: Early detection and classification of live bacteria using time-lapse coherent imaging and deep learning
Source: Light Sci Appl. 2020 Jul 10;9:118. doi: 10.1038/s41377-020-00358-9 (PMC7351775; doi:10.1038/s41377-020-00358-9)
Supplement: Supplementary file 1 — Supplementary Information [file 41377_2020_358_MOESM1_ESM.pdf]

Supplementary Information

Early detection and classification of live bacteria using time-lapse coherent imaging and deep learning

Hongda Wang[1,2,3†], Hatice Ceylan Koydemir[1,2,3†], Yunzhe Qiu[1,2,3†], Bijie Bai[1,2,3], Yibo Zhang[1,2,3], Yiyin Jin[1], Sabiha Tok[1,2,3,4], Enis Cagatay Yilmaz[1], Esin Gumustekin[5], Yair Rivenson[1,2,3], Aydogan Ozcan[1,2,3,6,*]

[1]Electrical and Computer Engineering Department, University of California, Los Angeles, CA, 90095, USA

[2]Bioengineering Department, University of California, Los Angeles, CA, 90095, USA

[3]California NanoSystems Institute, University of California, Los Angeles, CA, 90095, USA

[4]Department of Biophysics, Istanbul Medical Faculty, Istanbul University, Istanbul, 22000, Turkey

[5]Department of Microbiology, Immunology, and Molecular Genetics, University of California, Los Angeles, CA, 90095, USA

[6]Department of Surgery, David Geffen School of Medicine, University of California, Los Angeles, CA, 90095, USA.

†Equal contributing authors.

*Corresponding author: ozcan@ucla.edu

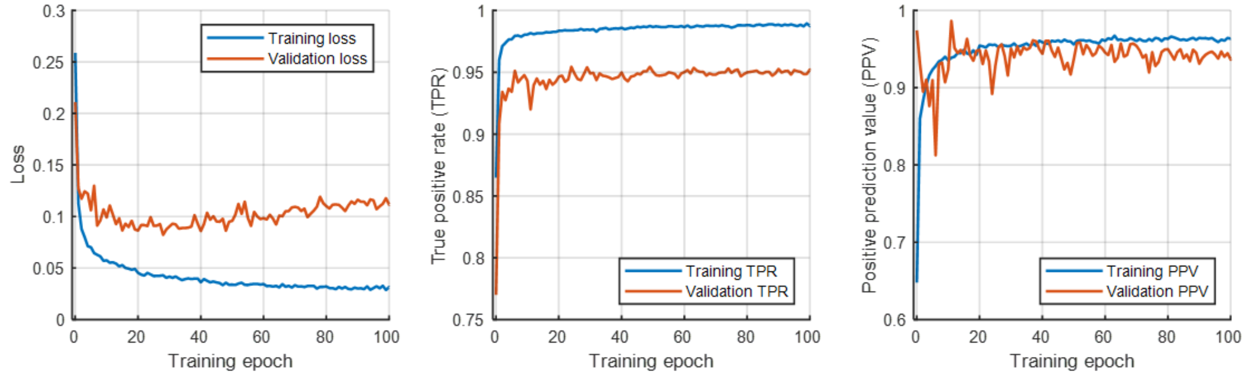

Figure S1. Training loss, true positive rate (TPR), and positive prediction value (PPV) curves of the neural network model for colony growth detection.

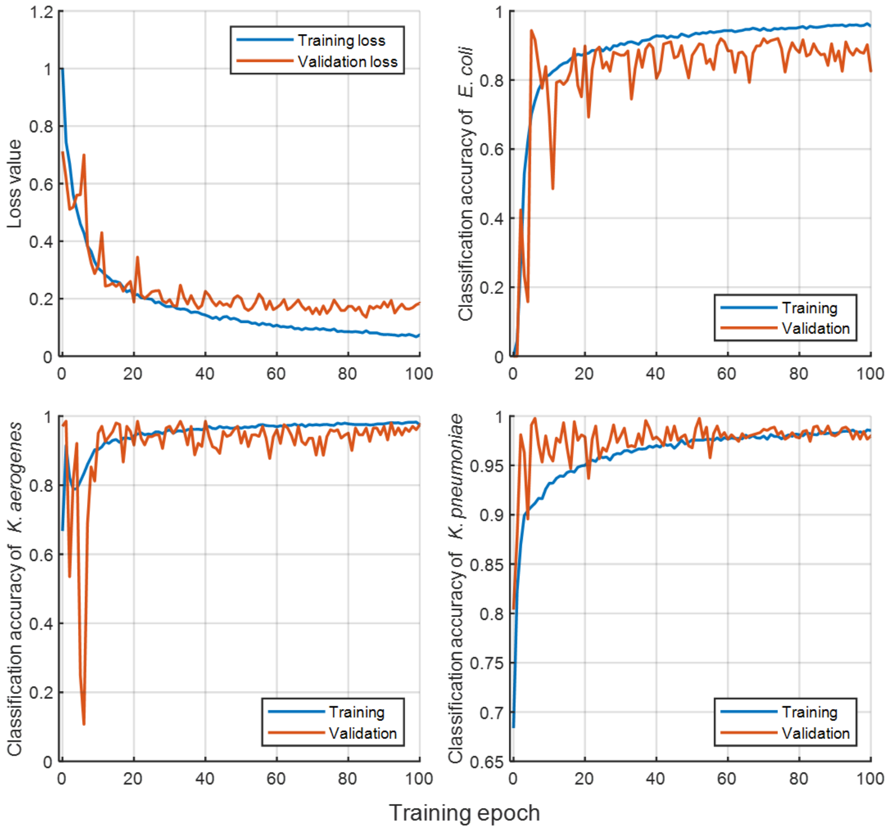

Figure S2. Training loss and classification accuracy curves of the neural network model for colony species classification.

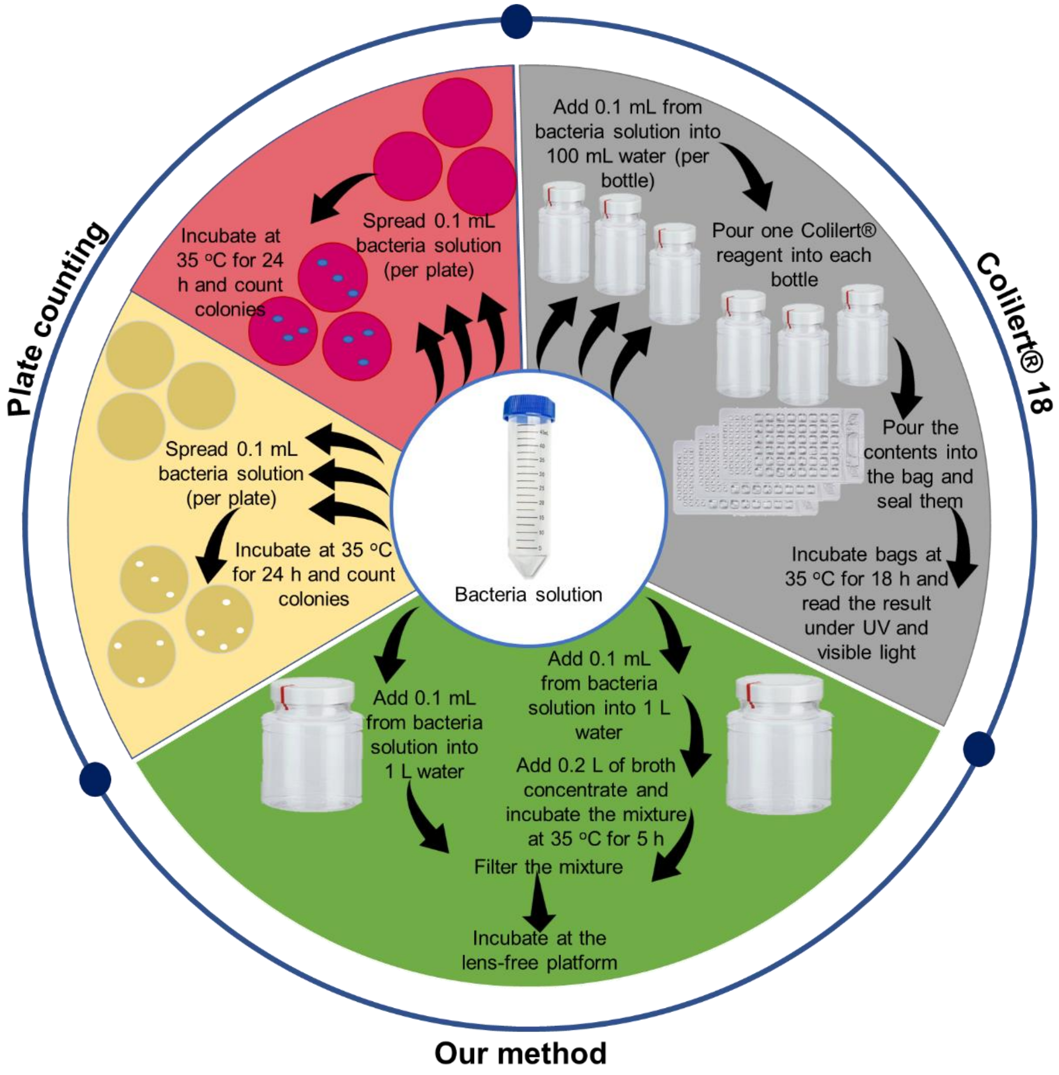

Figure S3. Schematics comparing the major steps involved in each one of the three different methods analyzed in this work.

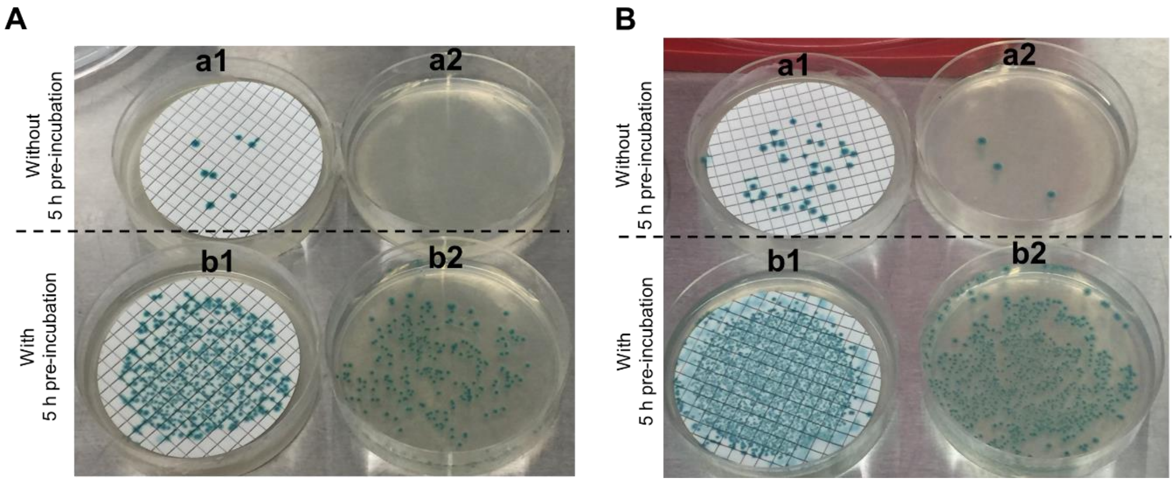

Figure S4. Example photos of CHROMagar™ ECC plates for two different bacterial concentrations of (A) 37.9± 5.6 CFU L^{-1}, (B) 160 ± 21.3 CFU L^{-1}. a1) No pre-incubation, after transferring bacteria from filter membrane to the agar plate and incubation at a benchtop incubator for 24 h. a2) No pre-incubation, transferred bacteria from the filter membrane in the photo of a1, incubated at the lens-free imaging setup for 24 h. b1) 5 h pre-incubation, after transferring bacteria from filter membrane to the agar plate and incubation at a benchtop incubator for 19 h (total incubation time: 5 + 19 = 24 h). b2) 5 h pre-incubation, transferred bacteria from the filter membrane in the photo of a1, incubated at the lens-free imaging setup for 19 h (total incubation time: 5 + 19 = 24 h).

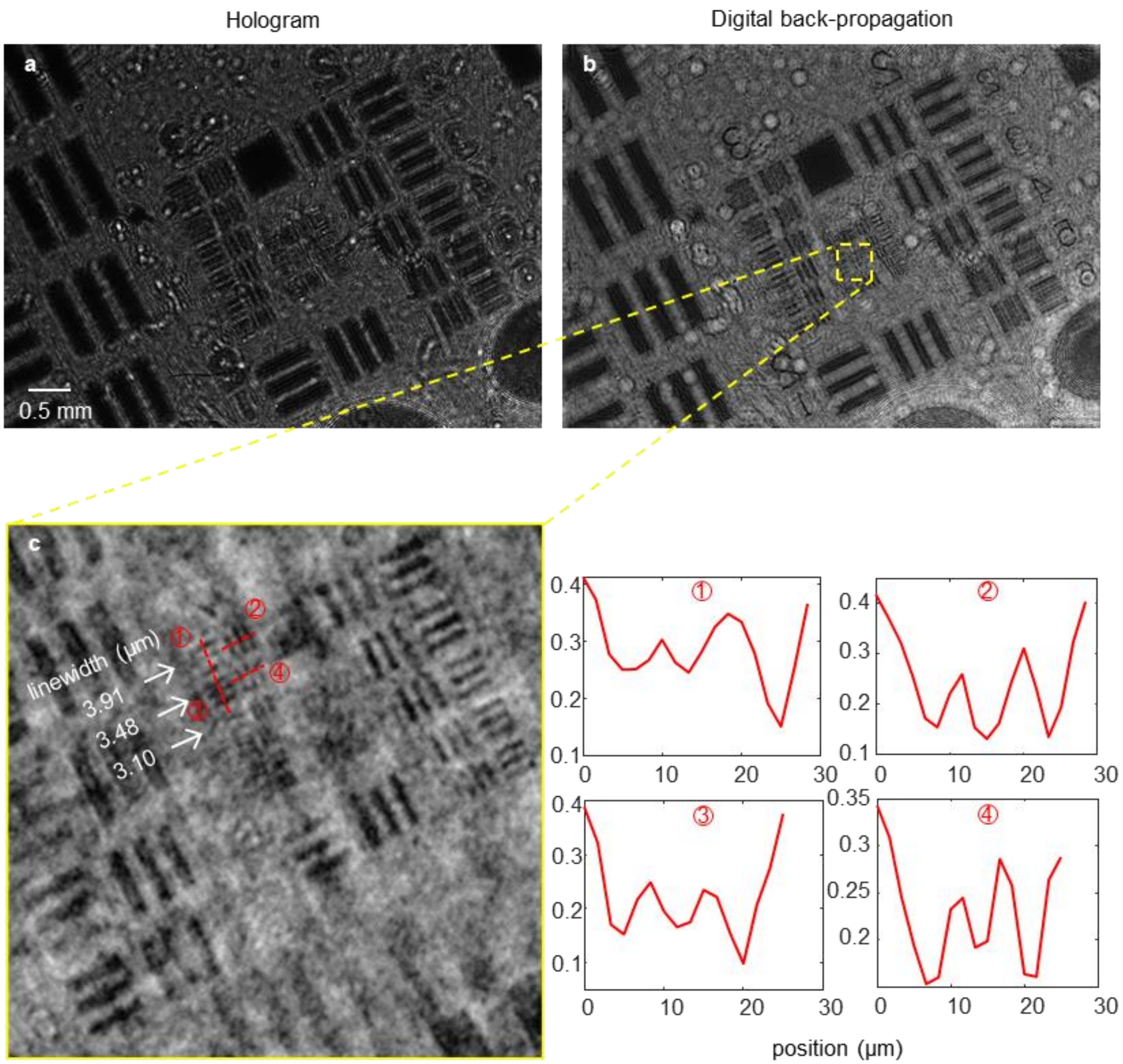

Figure S5. Resolution characterization of the lens-free bacterial colony detection system. (a) Raw hologram captured by the image sensor. (b) Digitally back-propagated hologram. (c) Zoomed-in region demonstrates a half-pitch resolution of ~3.5 µm.

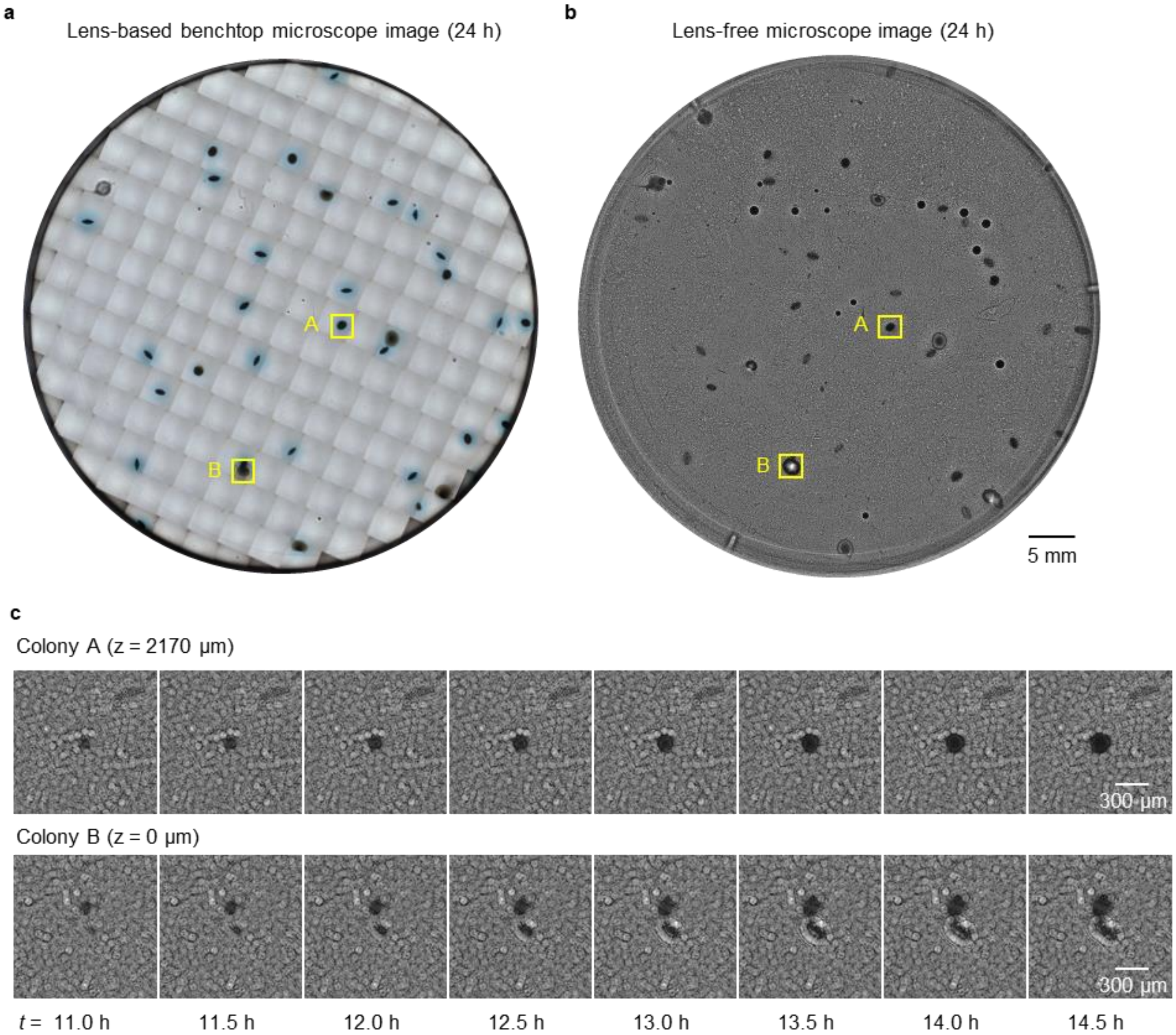

Figure S6. *E. coli* colonies grew at different depths within the 3D culture medium. (a) Image of the sample plate captured using a lens-based benchtop microscope after 24 hours of incubation and stitched by the microscope software. (b) Image of the sample plate captured using our lens-free microscope at 24 h of incubation. (c) Images of 2 colonies marked in (a) and (b) that grew at different depths, axially separated by ~2.17 mm.

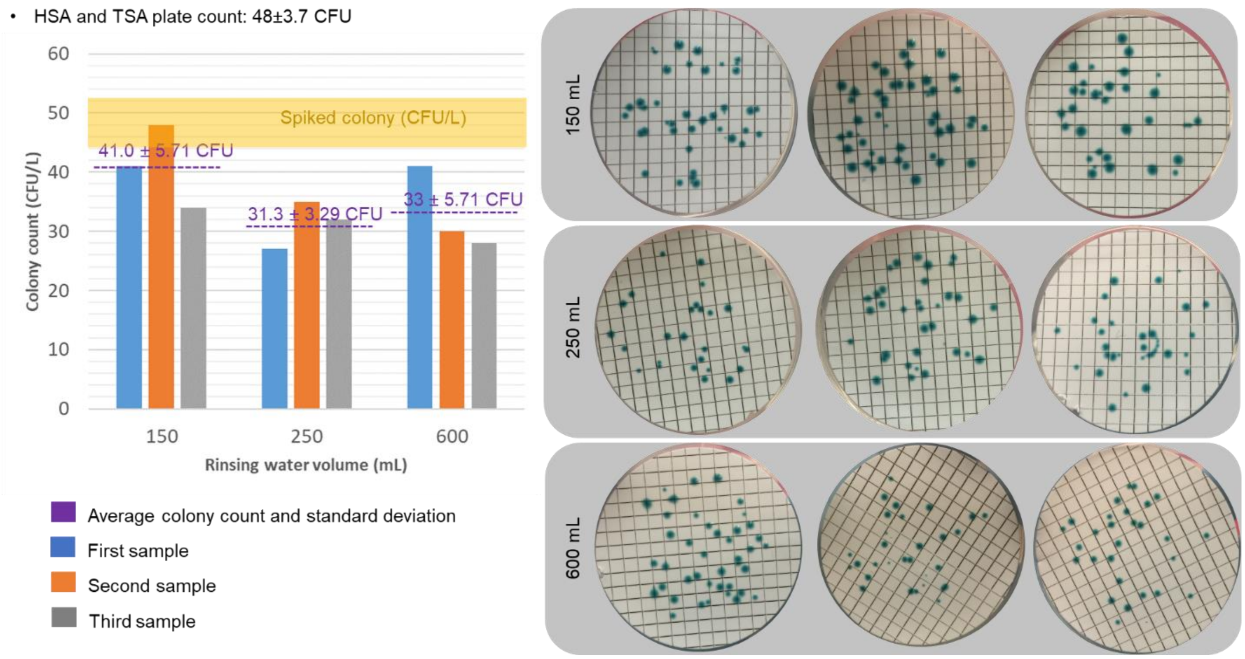

Figure S7. Colony counts obtained for optimization of the amount of water used for washing the sample container.

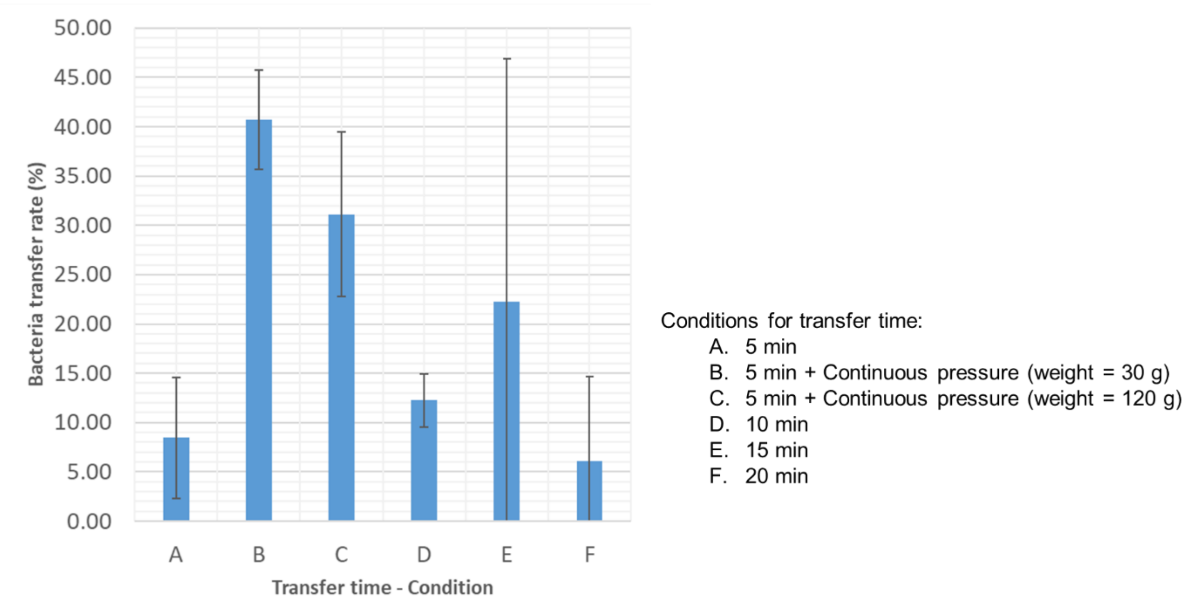

Figure S8. Bacteria transfer rate (%) obtained at different conditions of transfer time.

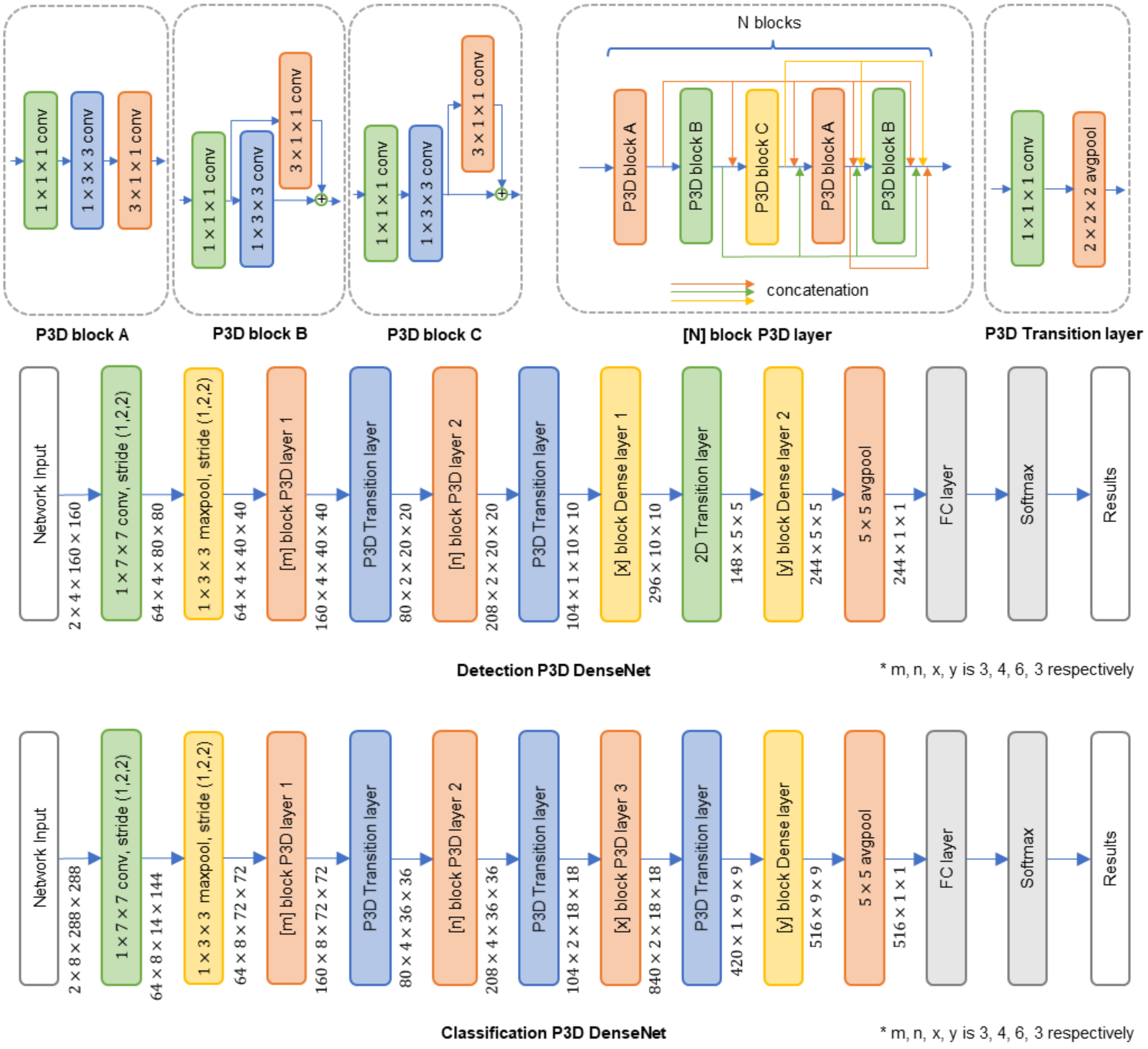

Figure S9. Schematic of pseudo-3D (P3D) DenseNet models for the detection and classification of growing colonies using the lens-free imaging system. The detection neural network model has 1.21×10^6 trainable parameters. The classification neural network model has 1.36×10^6 trainable parameters.

Table S1. Colony counts of some *E. coli* spiked samples in comparison to Colilert®-18 and plate counting.

Colilert®-18					Plate counting (TSA plates)					Plate counting (ECC ChromoSelect Selective Agar plates)				
R1*	R2*	R3*	Average	Std. deviation	R1†	R2†	R3†	Average	Std. deviation	R1†	R2†	R3†	Average	Std. deviation
172.3	172.3	135.4	160.00	21.30	169	162	198	176.33	19.09	164	137	140	147.00	14.80
11	17.3	20.1	16.13	4.66	15	18	14	15.67	2.08	17	13	17	15.67	2.31
225.4	166.4	228.2	206.67	34.90	228	260	246	244.67	16.04	245	241	221	235.67	12.86
8.6	8.5	12.1	9.73	2.05	4	4	5	4.33	0.58	2	5	11	6.00	4.58
37.9	43.5	32.3	37.9	5.6	52	37	30	39.67	11.24	35	28	36	33.00	4.36
3.1	1	<1	2.05	1.48	3	1	0	1.33	1.53	3	3	2	2.67	0.58
107.6	113.7	101.7	107.67	6.00	76	116	99	97.00	20.07	150	134	123	135.67	13.58
172.3	210.5	121.1	167.97	44.86	165	165	141	157.00	13.86	169	171	164	168.00	3.61

R is for replicate sample
* CFU per 100 mL
† CFU per 0.1 mL

Table S2. Comparison of our device against a scanning bright-field microscope for imaging of an agar plate (60 mm diameter).

Configuration	This work	Bright-field microscope (4 ×/0.1 NA objective lens)
Field of view (FOV) per image (mm^2)	29.4	14.4
Total FOV scanned (mm^2)	~3491	~2977
Total imaging time per agar plate (min)	~1.5	128
Effective pixel count (million)	570	435
Observation depth (µm)	> 20,000	3,000 (with 20 µm accuracy) *

* A larger focusing range and/or a higher accuracy would further increase image capture time.
